# Obstetric Aid

**Published:** 1874-08

**Authors:** 


Obstetric Aid.—The best contrivance is one which I shall venture to call my own, though it may, very likely, have been suggested before. It acts on the principle of directing muscular action, and consists of a sheet twisted loosely in the form of a rope, and tied together at the ends. Put the feet into the loop at the lower end, and push; grasp the other end with the hands and pull; the power exerted in this way is indefinite; it is the gymnastic paradox of trying to lift oneself, and may be practiced, if desired, till the sheet or back gives way. Its effect in labor is surprising, and is immensely appreciated by patients. It brings the body muscles into play. It relieves that distressing sense of helplessness, which all women feel, by enabling them to help themselves. It shortens labor. It saves the use of instruments. Allow me to recommend it to your readers.—*Boston Medical and Surgical Journal.*

